# Risk Assessment and Fertility Counseling for Hereditary Gynecological Cancer Syndromes

**DOI:** 10.1002/cam4.71206

**Published:** 2025-09-03

**Authors:** Mei Zhao, Xiao‐ming Teng, Qiang Yan, Fan Hao

**Affiliations:** ^1^ Reproductive Medical Center Shanghai First Maternity and Infant Hospital, School of Medicine, Tongji University Shanghai P.R. China

**Keywords:** autosomal dominant inheritance, cervical cancer, endometrial cancer, fertility preservation, genetic counseling, hereditary gynecological cancers, ovarian cancer

## Abstract

**Objective:**

To review the genetic basis, clinical characteristics, and management strategies of hereditary gynecologic cancers associated with hereditary cancer syndromes.

**Methods:**

Literature on germline mutations, inheritance patterns, clinical manifestations, and fertility preservation strategies was reviewed.

**Results:**

Germline pathogenic mutations, predominantly inherited in an autosomal dominant manner, increase susceptibility to gynecologic tumors with varying risks. Genomic sequencing has facilitated identification of high‐risk individuals, underscoring the importance of tailored prevention, early detection, and treatment. Standardized counseling supports risk assessment, fertility preservation, and the formulation of individualized management strategies.

**Conclusion:**

Comprehensive genetic counseling and precision‐based approaches are essential for effective prevention, diagnosis, and treatment of hereditary gynecologic cancers, while also addressing fertility preservation in affected patients.

## 1 Introduction

Hereditary cancer syndromes (HCS) are characterized by pathogenic mutations in one or more genes that cause tumors to develop in one or more organs of an individual, and the mutated gene(s) can be inherited from generation to generation in the family line, most often in an autosomal dominant manner, and account for about 5% to 10% of all tumors [1]. Hereditary tumors make up a small percentage of the population. However, their susceptibility can have a significant impact on family members. More than 50 different HCS have been identified, and this number will continue to increase over the coming [2]. The most common HCS related to gynecologic tumors include hereditary breast and ovarian cancer (OC) syndrome (HBOC), Lynch syndrome (LS), Cowden syndrome (CS), Peutz–Jeghers syndrome (PJS), and hereditary leiomyomatosis and renal cell carcinoma syndrome (HLRCC) [3]. With advancement in testing technology, more people can be expected to be diagnosed with HCS [4] and that the population of people who develop hereditary gynecologic cancers (HGC) will gradually increase. Fertility preservation (FP) is particularly important considering the obvious deleterious effects of gynecologic oncogenesis on female fertility and the shift in the goal of modern oncology from maintaining the life of the patient to optimizing her quality of life.

Obstetrician–Gynecologists and reproductive medicine physicians need to be knowledgeable about hereditary gynecologic neoplasms and strategies for their prevention, and provide counseling related to gynecologic oncology for high‐risk patients, including FP and preimplantation genetic testing (PGT), and develop a treatment plan.

### 1.1 Hereditary Gynecological Cancers Syndrome

Three common gynecologic malignancies, such as ovarian, endometrial, and cervical cancers, may be part of specific HCS. Therefore, gynecologists need to have a proper insight into HCS. HBOC, LS, CS, PJS, and HLRCC are associated with hereditary ovarian, endometrial, and cervical cancers, all of which are inherited in an autosomal dominant manner, and HBOC and LS are the most common HGCS. HBOC and LS are the most common HCS in the clinic, and the PJS population has an increased risk of developing minimal deviation adenocarcinoma (MDA) of the uterine cervix, but it is rare in the clinic. This chapter summarizes the clinical features for hereditary gynecological cancers presented in Table 1.

**TABLE 1:** Main characteristics of the most common hereditary gynecological cancers.

Syndrome	Gene mutations	Related gynecological cancers	Common pathological types	Other nongynecological cancers
Cowden syndrome	PTEN	Endometrial cancer	Endometrioid carcinoma	Breast cancer, Follicular thyroid cancer, gastrointestinal hamartomatous polyps, ganglioneuroma
Hereditary breast and ovarian cancer syndrome (HBOC)	BRCA‐1 and BRCA‐2	Ovarian cancer	High‐grade serous carcinoma Endometrioid carcinoma	Breast cancer, prostate cancer, pancreatic cancer
Hereditary leiomyomatosis and renal cell carcinoma syndrome	FH	Hysteromyoma	Uterine leiomyoma	Renal cancer
Lynch syndrome (LS)	MLH1, MSH2, MSH6, PMS2	Endometrial cancer	Ovarian endometrioid carcinoma Clear cell carcinoma, Carcinosarcoma	Colorectal cancer, gastric cancer, hepatobiliary tract cancer, small intestine cancer, ureteral cancer, bladder cancer, prostate cancer, breast cancer, pancreatic cancer
		Ovarian cancer	Non‐serous	
Peutz–Jeghers syndrome (PJS)	STK11/LKB1	Non‐epithelial ovarian tumor	Sex cord tumor with annular tubules (SCTAT)	Colorectal cancer, gastric cancer, small owel cancer, pancreatic cancer
		Cervical cancer	Gastric‐type endocervical adenocarcinoma (G‐EAC)	

### 1.2 BRCA‐Related Breast/Ovarian Cancer Syndrome (Hereditary Breast and Ovarian Cancer: HBOC)

HBOC, which is associated with germline mutations in the breast cancer 1 (BRCA1) and breast cancer 2 (BRCA2) genes, is responsible for breast cancers, OC, pancreatic, and prostate cancer [5]. The cumulative risk of developing breast and OC at the age of 80 for BRCA1 mutation carriers is 72% and 44%, and for BRCA2 mutation carriers is 69% and 17%, respectively [6]. More than 70% of BRCA‐associated OC are high‐grade plasma adenocarcinomas, and the rest are endometrioid carcinomas, whereas disseminated OC exhibit a variety of histologic types [7].

### 1.3 Lynch Syndrome

Lynch syndrome (LS) is an autosomal dominant disorder caused by a germline pathogenic variant in one of four DNA mismatch repair (MMR) genes (MLH1, MSH2, MSH6, PMS2) [8]. Individuals with LS are susceptible to colorectal cancer (CRC), endometrial cancer (EC), OC, and other cancers that vary based on MMR gene and age. An international multicenter prospective observational study including 6350 carriers of MMR gene variants showed that female MLH1, MSH2, and MSH6 carriers have a rapidly increasing risk of gynecological cancers from the age of 40 years. Heterozygous carriers of PMS2 variants do not have an increased risk of CRC, EC, or OC before age 50, and the risk may increase only slightly at older ages [9]. Recently, a report from the Prospective LS Database showed that at age 75 years, gynecologic cancers were more prevalent than CRC in MSH2, MSH6, and PMS2 mutation carriers, with cumulative incidence rates of 53.3%, 49.6%, and 23.3%, respectively [10]. The prevalence of LS mutations varies from person to person, and the risk of developing different tumors varies, with LS‐associated EC having a lifetime risk of up to 60%, and OC a lifetime risk of 24% [11].

LS‐associated EC has a younger age of onset than patients with sporadic EC, low BMI. Pathohistological manifestations are diverse, including endometrioid carcinoma, plasma carcinoma, clear cell carcinoma, and other highly malignant tumors, but endometrioid adenocarcinoma is the most common. The lower part of the uterine body is the most common site of carcinoma, but rarely involves the cervix [12]. A clear age of onset may provide a theoretical basis for developing individualized preventive measures for patients with LS.

### 1.4 Cowden Syndrome

Cowden syndrome is an autosomal dominant disorder caused by germline mutations in the PTEN gene. PTEN is expressed in all types of cells and is one of the most mutated oncogenes. It is characterized by multiple misshapen tumors and a high risk of breast, thyroid, kidney, and ECs [13]. The lifetime prevalence of breast cancer [14] and EC in CS patients is 85% and 28%; its onset begins at age 25 and rises to 30% by age 60, with the earliest reported case at age 3 years [14]. Most cases occur before the age of 50 years [15]. The majority of EC associated with CS are endometrioid adenocarcinomas (42%), some are plasma adenocarcinomas or clear cell carcinomas (5%), and a small percentage are mucinous adenocarcinomas (0.3%) [13].

### 1.5 Peutz–Jeghers Syndrome

Peutz‐Jeghers (PJS) syndrome is an autosomal dominant disorder closely associated with mutations in the STK11/LKB1 gene. It is closely associated with the development of a variety of malignant tumors, most commonly colorectal malignancies, followed by malignant tumors of the stomach, small intestine, duodenum, and pancreas, and with its associated gynecologic system tumors, mainly ovarian circumscribed tubulo‐testicular gonadotropic tumors (SCTAT) and gastric‐type endocervical adenocarcinoma (G‐EAC) [16]. The cumulative lifetime risk is18% to 21% and 10%, respectively [17]. Patients with PJS who develop G‐EAC have an early age of onset, with a mean age of 33 years, and are difficult to diagnose and have a poor prognosis, with normal cervical cytologic screening results in 50% of patients [17].

Additionally, compared with the West, there are significant differences in the tumor profiles of PJS patients in China and Japan, especially in the incidence of gynecologic tumors, with G‐EAC being the most common, and the tumors are diagnosed significantly earlier in female patients than in men [18].

### 1.6 Hereditary Leiomyomatosis and Renal Cell Carcinoma Syndrome (HLRCC)

HLRCC is a rare autosomal dominant disorder. The disease is caused by a germline heterozygous variant of the fumarate hydratase (FH) gene, which leads to abnormalities in the body's metabolism, resulting in a range of clinical conditions including multiple cutaneous smooth muscle tumors, uterine fibroids, and a higher risk of renal cell carcinoma (RCC) [19]. In patients with HLRCC, multiple uterine fibroids are a notable clinical manifestation, and these fibroids tend to be numerous, large, and have a high rate of recurrence after culling. Due to metabolic abnormalities caused by FH gene variants, uterine fibroids in patients with HLRCC may have a greater proliferative capacity and a higher risk of malignancy [20]. Most patients with HLRCC begin to develop multiple, symptomatic fibroids around the age of28 to 30 years [21].

## 2 Risk Assessment for Hereditary Gynecologic Cancer

**FIGURE 1:**
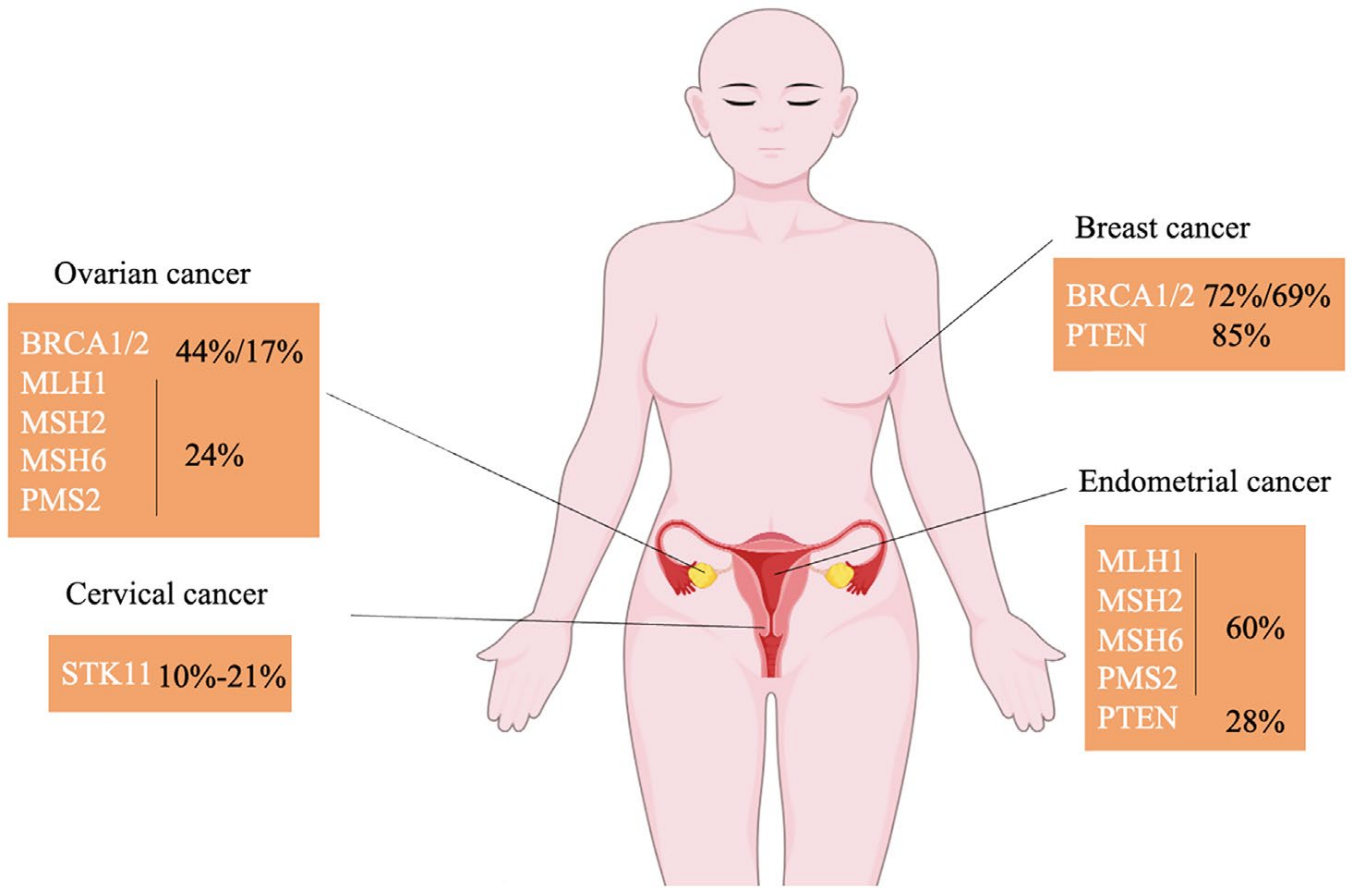
Prevalence of pathogenic variants in breast, ovarian, endometrial, and cervical cancers.

Gynecologists need to have a proper insight into HCS. The prevalence of pathogenic variants in gynecological cancers and breast cancer is shown in Figure 1. Hereditary tumor risk assessment is an effective way to identify individuals at high risk for developing a particular tumor and includes personal and family history, as well as pathology, imaging, and other tumor risk factors. Typical clinical features of hereditary cancers are as follows [1]: (1) Diagnosis of breast, ovarian, or rectal cancer at an early age or < 50 years. (2) Multiple tumor types in the same family member. (3) Multiple primary tumor foci in the same family member, especially in the same organ (e.g., breast or colon). (4) Multiple close family members (e.g., mother, daughter, or sister with breast cancer) with the same type of tumor. (5) Certain specific benign diseases such as skin diseases and developmental abnormalities of the skeletal system. (6) Specific types of tumors, such as triple‐negative breast cancer (estrogen and progesterone receptor‐negative and HER2 expression deficiency) and plasma ovarian epithelial, fallopian tube, and peritoneal carcinomas suggestive of HBOC, and rectal cancers with DNA MMR defects and EC with DNA MMR defects suggestive of LS. The purpose of genetic risk assessment is to identify individuals who may have HCS and who may benefit from genetic testing, additional screening, or preventive medical intervention.

Obstetricians and gynecologists should perform the HGC risk assessment. If the results of the assessment indicate a high risk for hereditary tumors, referral to an oncogeneticist for more in‐depth risk assessment and counseling or even genetic testing and appropriate tumor screening or risk reduction interventions is recommended.

With the development of genetics technology, cancer risk assessment can be performed through genome profiling [22].

For patients with suspected hereditary tumors, the key to exactly how to choose a genetic testing program is an adequate family history and personal history assessment. Medical interventions for these patients carrying mutated genes should be considered based on the results of genetic risk assessment. For example, microsatellite instability (MSI) testing of tumor tissue can reveal the possibility of hereditary cancers. LS was identified in 16.3% of patients with MSI‐high tumors. Broad MMR gene deletions require further evaluation of methylated promoters to determine whether they are systemic or germline mutations. Next‐generation sequencing (NGS), or multigene panel testing (MGPT), allows for the simultaneous detection of multiple genes and improves the diagnosis of disease in populations susceptible to inherited cancers [8, 23].

## 3 Clinical Management for Carriers of Hereditary Gynecologic Cancer Pathogenic Variants

Carriers of pathogenic variants associated with HGC should undergo timely screening for tumors to enable early lesion detection and reduce patient mortality. Commonly employed clinical screening tools include gynecological ultrasound, CA‐125 testing, endometrial biopsy, and cervical cytology smears. Additionally, medical interventions such as pharmacological prevention and risk‐reducing surgery (RRS) can effectively mitigate the risk of HGC.

### 3.1 Screening

Hereditary Ovarian Cancer. There are currently no effective screening tools for early detection. However, for individuals identified as high‐risk by the Genetic Counseling Institute, it is recommended to undergo vaginal ultrasound and serum CA‐125 testing every 6 months starting at age 35. These ultrasounds should be conducted by experienced sonographers in tertiary care or high‐volume centers [24]. Despite these measures, screening plays a limited role in the early detection of OC.

### 3.2 Risk‐Reducing Medicines

Oral contraceptive pills (OCPs) protect against ovarian and EC. OCPs use not only reduces the risk of OC by 50% and EC by 53% in the general population, but also reduces the risk of EC by 64% in obese women [25]. The protective effect continues for decades after the pill is stopped [26]. However, it is controversial whether OCPs increase the risk of breast cancer in people with BRCA mutations as the duration of dosing increases [27, 28]. Pooled observational data from four recent prospective cohort studies suggest that OCPs increase the risk of breast cancer in BRCA1 mutation carriers, especially if used for longer durations. However, this has not been validated in case–control studies in the BRCA2 mutation population [29]. So, it is prudent to weigh the risks and benefits for each individual and to achieve full informed consent for the use of OCPs in people with BRCA1 mutations who have no history of breast cancer.

### 3.3 Risk‐Reducing Surgery

Prophylactic interventions in hereditary gynecological cancer syndromes, such as prophylactic mastectomy, salpingo‐oophorectomy, and chemoprevention, are subject to ongoing debates. One of the main debates regarding prophylactic surgeries is the balance between the potential benefits and the associated risks and quality‐of‐life implications.

#### 3.3.1 Risk‐Reducing Salpingo‐Oophorectomy (RRSO)

RRSO is the most effective method available to reduce the risk of OC, and implementation of RRSO reduces the risk of gynecologic tumor by 80% to 90% and reduces all‐cause mortality by 77%. The timing of RRSO is based on the age of the patient and the mutation. RRSO is recommended for BRCA1 mutation carriers aged 35 to 40 years and BRCA2 mutation carriers aged 40 to 45 years [24].

#### 3.3.2 Prophylactic Salpingectomy

Ovary high‐grade plasmacytoid carcinoma originates in the fallopian tubes, and prophylactic salpingectomy may delay the need for oophorectomy in women with high genetic risk [30]. At this point, an experienced sonographer can differentiate between benign and malignant ovarian tumors to avoid unnecessary surgery [24]. Although several studies have demonstrated the safety and feasibility of this procedure, long‐term prospective studies [31] are needed to determine its effectiveness in reducing the risk of OC and improving menopause [32].

For gynecologic cancers in LS, hysterectomy and bilateral salpingo‐oophorectomy (hyst‐BSO) at age 40 is recommended for MSH2 mutation carriers. For MLH1 and MSH6 mutation carriers, a new 2‐stage surgical approach (i.e., hysterectomy and salpingo‐oophorectomy at age 40 years and delayed oophorectomy at age 50 years) may be an alternative to hyst‐BSO at age 40 years to avoid early menopause, whereas for individuals with the PMS2 mutation, findings suggest that hyst‐BSO can be delayed until age 50 years [33].

In conclusion, the ideal strategy for balancing tumor prevention and FP in the HGC population is risk‐reducing surgery after completion of childbearing.

Established FP methods for women, including embryo, oocyte, and ovarian tissue cryopreservation (OTC), and conservative gynecologic surgery [34]. The only established FP method applicable to prepubertal females is OTC. In vitro maturation (IVM) of oocytes, artificial ovaries, and induced multifunctional stem cell differentiation can be some of the emerging methods. Gonadotropin‐releasing hormone agonists (GnRHa) do not replace existing FP methods but may be used as an adjunctive therapy to female patients with breast cancer.

## 4 Established FP

### 4.1 Embryo and Mature Oocyte Cryopreservation

Ovarian reserve testing, including antral follicle count (AFC) or anti‐mullerian hormone (AMH) serum level testing, should be recommended for all patients prior to treatment to predict ovarian response during stimulation to facilitate individualized FP strategies for patients [35].

Embryo cryopreservation technology, as the most mature and stable method of female FP, has demonstrated significant clinical application value in the hereditary gynecological tumor patient population. According to the latest meta‐analysis data, breast cancer patients (including hereditary BRCA mutation carriers) who underwent embryo cryopreservation prior to tumor treatment had cumulative live birth rates ranging from 33% to 63.2%, a success rate close to that of conventional assisted reproduction techniques. A meta‐analysis that included nine studies of pregnancies from frozen embryo transfers in breast cancer patients found that the clinical pregnancy rate and the live birth rate reached 50% (95% CI: 35–65) and 33% (95% CI: 22–46), respectively [36].

For single women with HCS, elective oocyte cryopreservation may be considered before the possible onset of cancer. In addition, the lack of time pressure allows for multiple cycles of oocyte retrieval, which in turn allows for the cryopreservation of a greater number of oocytes. This approach could increase the future chances of pregnancy in women with HCS. Pooled data from 10 studies reporting on the live birth rate resulting from planned oocyte cryopreservation found that women aged ≤ 35 years or younger had a higher live birth rate, which is more than twice as high compared to women over 40 years of age [37]. Whether patients with childhood‐onset cancer can benefit from the procedure is controversial because the cancer develops too early. An article reports the first successful oocyte cryopreservation in a prepubertal girl with Turner syndrome mosaicism. This heralds that oocyte preservation is technically feasible in adolescent patients [38]. This finding highlights the potential of oocyte cryopreservation as an adjunct to optimize FP protocols in premenarcheal girls. Both oocyte and embryo cryopreservation require one cycle of gonadotropin‐based controlled ovarian stimulation (COS). Although assisted reproductive technology has not been found to increase the risk of disease in patients with estrogen receptor (ER)–positive tumors [39], it is prudent to keep estradiol levels under strict control during assisted conception, with as minimal a rise as possible, and the option is an aromatase inhibitor (letrozole) combined with a gonadotropin stimulation regimen. In a multicenter randomized controlled study, 162 breast cancer patients were randomized into three groups: ovulation induction combined with tamoxifen, ovulation induction combined with letrozole, and conventional ovulation induction. There was no statistically significant difference in the number of oocytes or embryos in their three groups [40]. And there was no difference in overall survival between women with breast cancer who received COS and those who did not [41]. Although fertility treatments may increase OC risk [39], limited data confirm that ART does not increase the risk of OC in BRCA mutation carriers, at least in the short term [42]. Similarly, patients exposed to ART after completion of cancer‐directed treatments showed an improved recurrence ratio compared to those who did not undergo ART, with no difference in event‐free survival observed [43].

### 4.2 Ovarian Tissue Cryopreservation (OTC)

Ovarian tissue cryopreservation (OTC) is an option for adolescents or for those who are unable to undergo oocyte or embryo cryopreservation for objective reasons [44]. Originally categorized as experimental, OTC has now been considered a clinical standard practice in Europe [45]. Research has shown that endocrine recovery is achieved in more than 95% of adult cases, with a pregnancy rate of 40% [46].

However, there are two major difficulties in the application of OTC transplantation: firstly, follicle loss, the main causes of which include cryo‐cryogenic damage and slow revascularization and insufficient perfusion after transplantation; and secondly, there is a risk of primary tumor cell reimplantation after transplantation [46]. Therefore, ovarian tissue freezing and transplantation should be used with caution in patients with ovarian malignancies. OTC protocols for adults are not fully applicable to children, and targeted freezing and transplantation protocols must be developed based on the structural and functional characteristics of children's ovarian tissue, due to the vast differences in ovarian tissue characteristics between children and adults. OTC also requires large sample studies to validate its effectiveness in restoring fertility function and long‐term offspring safety. There have been no reported cases of live births after OTC in patients with TS, and most of the patients who underwent OTC were not yet of childbearing age or had no need for childbearing for the time being.

### 4.3 Preimplantation Genetic Testing (PGT)

Women who carry pathogenic mutations are at higher risk of developing cancer, and such patients should receive genetic counseling related to PGT, which begins with obtaining embryos through in vitro fertilization techniques, biopsying the embryos (often at the blastocyst stage), and screening for embryos that do not carry pathogenic mutations for transfer, preventing vertical inheritance of the disease in couples with inherited disorders [47]. For clear single‐gene diseases and breast cancer caused by certain highly epigenetic susceptibility genes, such as the BRCA1 pathogenic variant, genetic blockade of the corresponding diseases can be achieved by PGT‐M [48].

The field of genetic testing for hereditary gynecological cancer syndromes is witnessing continuous innovation, which holds great promise for improving diagnosis and patient management. Genome‐wide association studies (GWAS) of polygenic diseases have identified many associations between common variants and complex diseases. Polygenic risk scores (PRS) were developed to assess the impact of multiple risk SNPs associated with a certain phenotype on an individual. Then PGT‐P, also known as polygenic embryo screening (PES), can assess the risk of disease in embryos through multiple common variants and screen embryos with the lowest risk of disease for transfer, so as to reduce the risk of polygenic disease in the offspring from the source, thus realizing the first level of prevention and control of polygenic disease [49]. However, the GWAS cohort and PRS models are mostly constructed based on European populations, and the existing data models do not necessarily have the same predictive ability for all races, which will lead to a lack of sufficient basic data to support the application of PGT‐P. Moreover, PGT‐P gives a vague risk prediction, and it is also a major challenge for geneticists to understand the clinical significance of the risk and to explain the results clearly to couples undergoing PGT‐P [50]. Socially, ethically, and legally, PGT‐P may give rise to physiological harm and economic burden from unnecessary/excessive IVF treatment [51]. Therefore, the clinical application of PGT‐P is limited at present.

The convergence of artificial intelligence (AI) and PGT opens up new avenues to improve pregnancy outcomes, enable personalized treatment, and enhance reproductive health services. With the advancement of AI technology, multimodal learning is gradually becoming an important research direction. Barnes et al. [52] developed the STORK‐A system to predict embryo integrability by combining embryo morphological features, mother's age, and blastocyst score with an accuracy of 69.3% and an AUC of 0.761, and an accuracy of 77.6% in recognizing complex aneuploidies, with an AUC of 0.847. The study incorporates age, spatial features of embryo images, and other metrics into the AI model, which provides us with a new method for noninvasive preimplantation embryo screening by incorporating clinical features. This AI model may become a well‐established means of primary screening before performing PGT. However, the algorithms currently developed by AI lack the accuracy and robustness required to replace PGT in a clinical setting, and further improvement and validation in large‐sample clinical studies are still needed before clinical application.

### 4.4 GnRH‐a

Temporary ovarian suppression with gonadotropin‐releasing hormone (GnRH) agonists is an option for preserving ovarian function, but data on fertility outcomes (i.e., pregnancy rates) remain controversial [53]. Studies suggest that GnRH‐a may protect fertility by inhibiting gonadotropin secretion, inhibiting follicular development, and preventing follicle destruction by chemotherapeutic agents, as well as by decreasing uterine and ovarian perfusion and reducing chemotherapeutic agents' ability to reach ovarian tissue [54].

Several studies have been conducted to investigate whether the combination of GnRH‐a during chemotherapy has a protective effect on patients' fertility. It was found that in the group of combined application of GnRH‐a during chemotherapy, 65% to 75% of patients recovered normal menstruation and ovarian function, while in the group of chemotherapy alone, only 40% to 50% of patients recovered normal menstruation and ovarian function. Moreover, the natural pregnancy rate in the combination chemotherapy group was 11% to 35% higher than that in the chemotherapy alone group [55, 56]. In 2021, 281 patients with early‐stage breast cancer were included in a PROMISE‐GIM6 multicenter RCT, for whom early follow‐up showed that GnRH‐a facilitated menstrual recovery, and after a median follow‐up of 12.4 years, disease‐free survival (68.3%) and overall survival (83.2%) were found to be better in the combination chemotherapy group compared with the chemotherapy only group (68.5%, 82.3%). None of the differences were statistically significant. Forty‐three of the patients were tested for the BRCA gene, and in the BRCA population, the incidence of POI was 0% and 33% in the combination GnRH‐a group and the chemotherapy alone group, respectively [57]. Exploratory analyses of BRCA populations have also preliminarily shown positive implications for GnRHa ovarian protection. The use of GnRH‐a too early after oocyte collection may delay subsequent treatment due to its “flare‐up effect,” which may increase the risk of ovarian hyperstimulation syndrome (OHSS), while the use of long‐acting GnRHa has recently been shown to reduce the incidence of OHSS [58]. Therefore, the timing and type of GnRH‐a should be determined in consultation between oncologists and reproductive medicine physicians to assess the optimal and safe timing of GnRH‐a administration in these cancer patients with special needs.

Some studies have shown that: GnRH‐a is effective in hematology and breast cancer treatment, while for ovarian, endometrial, and cervical cancers, the evidence is still limited [54]. Prophylactic FP methods are still controversial and need to be confirmed by further clinical studies in the future.

### 4.5 Conservative Gynecologic Surgery

Radiotherapy‐induced damage to ovarian function is significantly associated with the irradiation dose. Specifically, a dose of 2 Gy results in a 50% reduction in follicular density, while doses exceeding 5 Gy can lead to amenorrhea in women over the age of 40. Consequently, ovarian translocation (OT) is considered a straightforward and effective strategy for FP in patients scheduled for pelvic radiotherapy, such as those with cervical cancer. Additionally, other FP methods, such as ovarian tissue cryopreservation, may be selected based on individual patient circumstances. The most prevalent postoperative complication is small bowel obstruction due to postsurgical adhesions [59]. A retrospective study of 1377 cervical cancer patients indicated that 61.7% of ovarian function was preserved following radiotherapy with OT [60]. However, a systematic review of 29 studies involving 1160 patients reported a high rate of preserved ovarian function: 93% (95% CI: 76–113) in the surgery ± brachytherapy (BR) group and 61% in the external beam pelvic radiotherapy (EBRT) ± BR ± surgery group [61]. Another retrospective study that included 59 adolescents and young adults found no significant difference in ovarian function recovery when OT was performed before or during puberty [62].

Uterine translocation surgery serves to preserve reproductive function in patients undergoing pelvic radiation therapy. A systematic review of the literature on uterine translocation examined 18 Cancer Patients, five of whom attempted conception and achieved successful pregnancies [63]. While OT has become a well‐established surgical technique for FP, uterine translocation remains in its nascent stages and is currently under exploration. OT and uterine translocation surgery represent a promising strategy for ovarian and fertility preservation; however, further rigorous scientific investigations are required to thoroughly assess fertility outcomes in young women and pediatric adolescents undergoing those treatments. Such studies will enhance our understanding of the efficacy of OT and uterine translocation surgery as a FP method, particularly in the context of early cancer treatment.

## 5 Emerging FP Techniques

### 5.1 In Vitro Maturation

Immature oocytes can be collected at any time during a woman's menstrual cycle, which is particularly suitable for patients with malignant tumors who cannot tolerate ovarian ovulation stimulation or who need to receive immediate radiotherapy or chemotherapy. Most of the data on IVM for FP comes from young women diagnosed with breast cancer. In a review that included 7711 oocytes from cancer patients (half of the breast cancer patients), IVM resulted in an overall oocyte maturation rate of 59.7% and the cryopreservation of 335 embryos and 2380 oocytes. IVM increased the number of mature oocytes after an IVF cycle, with similar rates of survival, meiotic resumption, and blastocyst formation when compared to the standard protocol [64].

Alternatively, IVM may serve as a complementary procedure to OTC. A systematic review study included 12 reviews that obtained 5724 immature oocytes by OTC, 33.8% of immature oocytes were successfully matured by IVM and 20.4% were successfully cryopreserved [65]. Gonadotropin improves oocyte maturation rates of IVM in premenarcheal girls undergoing FP, in which younger children benefit more [66].

IVM in patients with cancer is an emerging FP method. However, the technique of IVM is still in an immature stage and has not yet been widely used in clinical practice. One study found that equal halves of an entire ovary were processed using standard IVM or a biphasic IVM system (CAPA‐IVM). Better results, including the generation of frozen blastocysts, were obtained after using the CAPA‐IVM system [67]. So far, studies of oocytes and blastocysts produced after the CAPA‐IVM system did not display increased genetic aberrations [68]. CAPA‐IVM has shown promise in improving the competence of ovarian tissue oocytes in patients with gynecological malignancies. But the long‐term health of children born from these oocytes, as well as the potential impact of the in vitro maturation process on the offspring, is still unknown. Understanding these long‐term outcomes is crucial for providing accurate counseling to patients with HCS who are considering IVM as a FP option.

### 5.2 Other Techniques

A study utilized three‐dimensional (3D) culture methods for in vitro follicle culture and induction of follicle maturation [69]. Some studies have utilized artificial ovaries constructed with extracellular matrix skeletons or decellularized scaffolds to successfully restore ovarian function in animal studies [70]. Moreover, the addition of BM‐MSCs to the reconstructed ovaries increased the survival rate of oocytes [71]. However, the development and maturation process of human follicles is time‐consuming and complex, and further exploration is still needed for how to optimize the in vitro culture system to promote the coordinated development of the oocyte‐oocyte complex to achieve oocyte maturation and conceive offspring.

## 6 Summarize

Female FP techniques are undergoing continuous optimization and innovation, with significant advancements in the maturation of embryo and oocyte vitrification cryopreservation. OTC techniques require validation through larger cohort studies to assess their efficacy in fertility restoration and to ensure the long‐term safety of both patients and their offspring. Additionally, the longevity of transplanted ovarian tissue and the factors influencing it, such as transplantation site and follicular density, necessitate further clarification. Most emerging FP techniques remain in the experimental phase. Additionally, the gonadal toxicity associated with some newer oncological treatments, such as targeted therapies and immunotherapies, remains inadequately understood, particularly regarding their potential effects on ovarian reserve and fertility. Consequently, it is imperative to actively advance both basic and clinical research in female FP. This includes efforts to limit and enhance the use of gonadotoxic drugs, develop biomarkers for assessing fertility and oocyte quality, and refine and innovate FP methodologies. These endeavors should be prioritized as a critical area of investigation in the field of reproductive medicine moving forward.

## Author Contributions

**Mei Zhao:** conceptualization (equal), writing – original draft (equal), writing – review and editing (supporting). **Xiao‐ming Teng:** conceptualization (equal), visualization (supporting), writing – original draft (equal). **Qiang Yan:** investigation (equal), visualization (equal), writing – review and editing (supporting). **Fan Hao:** conceptualization (equal), investigation (supporting), project administration (lead), supervision (lead), validation (lead), writing – review and editing (lead).

## Conflicts of Interest

The authors declare no conflicts of interest. The figures in the manuscript were drawn in Figdraw.

## Data Availability

The authors have nothing to report.
